# Plasmin Generation Kinetics Reveal a Hyperfibrinolytic Phenotype in Patients with Pancreatic Ductal Adenocarcinoma

**DOI:** 10.1055/a-2925-6824

**Published:** 2026-09-15

**Authors:** Ruth A.L. Willems, Ilaria De Simone, Samia Tufaha, Joke Konings, Harmen Middelveld, Nicol Pepels-Aarts, Hugo Ten Cate, Bas De Laat, Mark Roest, Dana Huskens, Judith de Vos-Geelen

**Affiliations:** 1 Department of Functional Coagulation 580397 Synapse Research InstituteMaastrichtLimburgThe Netherlands; 2 Division of Medical Oncology and Vascular MedicineDepartment of Internal Medicine 199236 Maastricht University Medical CenterMaastrichtLimburgThe Netherlands; 3 199236 Thrombosis Expert Center Maastricht, Maastricht University Medical CenterMaastrichtLimburgThe Netherlands; 4 5211 CARIM, School for Cardiovascular Diseases, Maastricht UniversityMaastrichtLimburgThe Netherlands; 5 Department of Platelet Pathophysiology 580397 Synapse Research InstituteMaastrichtLimburgThe Netherlands; 6 Division of Medical OncologyDepartment of Internal Medicine 199236 Maastricht University Medical CenterMaastrichtLimburgThe Netherlands; 7 Division of Vascular MedicineDepartment of Internal Medicine 199236 Maastricht University Medical CenterMaastrichtLimburgThe Netherlands; 8 5211 GROW, Research Institute for Oncology and Reproduction, Maastricht University Medical CenterMaastrichtLimburgThe Netherlands

**Keywords:** cancer, fibrinolytic disorders, fibrinolysis inhibitors, thrombin, venous thrombosis

## Abstract

**Background** Thromboembolic complications are common in patients with pancreatic cancer, with up to 20% of patients developing venous thromboembolism. While hypercoagulation in pancreatic cancer has been widely studied, fibrinolysis is less well characterized. Plasmin generation (PG) is a novel approach to assess fibrinolytic activity and may provide new insights into thrombotic risk in this population. This study aimed to compare the PG profile of patients with pancreatic cancer with that of healthy individuals.

**Methods** This single-center cohort study included patients with advanced pancreatic cancer initiating chemotherapy at the Maastricht University Medical Center between 2022 and 2023. Plasma samples were obtained before chemotherapy ( *t*_0_ ) and 8 weeks later ( *t*_8_ ). PG and thrombin generation (TG) were measured; TG/PG ratios were calculated to evaluate the balance between coagulation and fibrinolysis. Thromboembolic events were recorded for 6 months.

**Results** In total, 18 patients with pancreatic cancer and 18 healthy subjects were included. At baseline, patients showed higher endogenous plasmin potential levels compared with controls, whereas PG remained elevated at *t*_8_ despite chemotherapy. PG lag time was prolonged in patients at *t*_8_ . Patients showed lower TG/PG ratios for endogenous thrombin/plasmin potential. Five patients (28%) developed thromboembolic events. Although PG parameters were not associated with thromboembolic events, patients who developed thromboembolic events showed lower TG/PG potential ratios.

**Conclusion** Patients with pancreatic cancer exhibited a disbalance in their PG profile, with a delayed but increased capacity to generate plasmin. Future studies are required to assess whether PG associates with thromboembolic disease and survival.

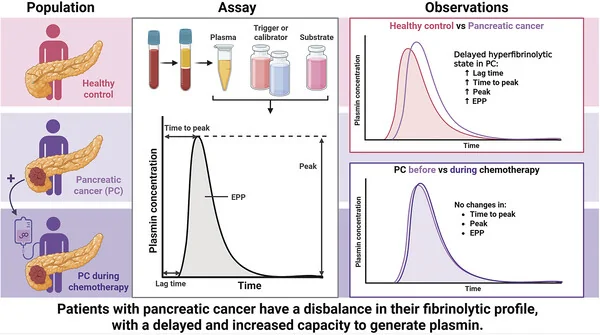

## Introduction

Thromboembolic disease is an important complication in patients with pancreatic cancer. Of all malignancies, pancreatic cancer carries the highest incidence of venous thromboembolic events (VTE). 1 2 About 20% of pancreatic cancer patients develop VTE, and the incidence of VTE has been growing in the last decade. 3 4 In addition to VTE, pancreatic cancer patients are at increased risk for arterial thromboembolisms (ATE). 5 6 7 Both VTE and ATE are associated with reduced overall survival rates in this patient population. 4 5 8

Following randomized trials, international guidelines recommend considering primary thromboprophylaxis in ambulatory cancer patients at high risk of VTE, most commonly identified using the Khorana score with a cutoff of ≥2 points. 9 10 11 12 13 Because pancreatic cancer alone confers two points, effectively all patients with pancreatic cancer are classified as high risk. 13 However, the Khorana score has limited discriminative ability within this population, as it poorly distinguishes between patients at intermediate and high risk of VTE. 14 15 Moreover, the absence of pancreatic cancer–specific biomarkers or risk assessment models hampers individualized risk stratification. Consequently, despite guideline recommendations, implementation of thromboprophylaxis remains limited in clinical practice. 9 11 16 17

Extensive research has explored the prothrombotic state of pancreatic cancer patients. 18 19 20 Recently, our research revealed that pancreatic cancer patients have an increased and delayed whole blood thrombin generation (WB-TG), with alterations in platelet and white blood cell (WBC) counts contributing to this dysregulation. Notably, a shorter lag time in WB-TG and enhanced platelet granule release predict thromboembolic event incidence. Platelets and WBCs appear to be a driving force in thrombosis development, highlighting the potential for whole blood hemostasis measurement to enhance thrombotic risk estimation in pancreatic cancer patients. 21

Coagulation is balanced by fibrinolysis. In patients with pancreatic cancer, elevated levels of fibrinolysis end products such as D-dimer and fibrin degradation products (FDP) are observed, reflecting both hypercoagulation and increased fibrinolysis. 22 23 In contrast, higher antigen levels and increased activity of plasminogen activator inhibitor-1 (PAI-1) are found in patients with pancreatic cancer. Higher levels of this key inhibitor of fibrinolysis might cause a hypofibrinolytic state and predispose patients with pancreatic cancer to VTE. 22 23 Although many assays are available to evaluate coagulation and prothrombotic factors, few are tailored for global fibrinolysis assessment. Studies using thromboelastography or clot lysis time (CLT) showed normal to hypofibrinolysis in pancreatic cancer. 24 25 26 Recent advancements in measuring plasmin generation (PG) kinetics, reflecting the ex vivo capacity of plasma to generate plasmin upon a standardized trigger, may provide useful insights into potential alterations in the fibrinolytic system. 27 28 29

In this pilot study, our objectives were 2-fold. First, we aimed to compare PG profiles, as measured with the calibrated automated kinetic PG assay, between pancreatic cancer patients and healthy individuals. Second, we aimed to explore associations between PG parameters and the occurrence of thromboembolic events in these patients.

## Materials and Methods

### Study Design and Study Population

The TROCOPAC study is a case–control proof-of-principle study including patients with metastatic and locally advanced pancreatic cancer (LAPC) from the outpatient clinic of the Maastricht University Medical Center (MUMC+). 21 The TROCOPAC study aimed to compare hemostasis markers between patients with pancreatic cancer and healthy controls. This study was assessed by the medical ethics committee of the MUMC+ and found not to be subject to the Medical Research Involving Human Subjects Act (METC 2021-3087). Patients and healthy volunteers gave full written informed consent, in accordance with the Declaration of Helsinki.

Eligible patients were recruited by their treating physician or oncology nurse. The inclusion criteria were: (1) age ≥18 years, (2) diagnosis of locally advanced or metastatic pancreatic cancer based on pathology, radiology, and/or biomarker tests, (3) before the first dose of a new chemotherapy regimen, and (4) written informed consent. Exclusion criteria were signs of a bacterial infection within 2 weeks prior to inclusion, a medical history of coagulation abnormalities, surgery within 1 month prior to inclusion, chemotherapy within 1 month prior to inclusion, and the use of anticoagulant drugs at a therapeutic dose. Patients were allowed to take antiplatelet medication and anticoagulant drugs in a prophylactic dose.

Healthy controls were recruited via a donor database of individuals who had expressed their willingness to donate blood for research (NL73968.068.20/METC 20-041). Healthy controls with a similar age and sex distribution to the patients were included.

### Time of Blood Collection and Follow-up

Blood samples were collected at baseline before chemotherapy initiation ( *t*_0_ ) and at 8 weeks of follow-up ( *t*_8_ ). The 8-week follow-up was chosen to assess treatment-related changes in PG and coincided with a routine treatment visit, as additional study-related blood sampling was not permitted in this study. At this time point, patients had generally completed approximately four cycles of FOLFIRINOX or two cycles of gemcitabine plus nab-paclitaxel. Thromboembolic events were defined as objectively confirmed ATE, including arterial myocardial infarction and cerebrovascular infarction, and all-type VTE, comprising symptomatic or incidental deep vein thrombosis, pulmonary embolism, and visceral vein thrombosis. The follow-up period started at time of blood sampling ( *t*_0_ ). Patients were prospectively followed for 6 months after study inclusion or until the occurrence of death or loss to follow-up.

### Blood Collection and Plasma Preparation

Blood from patients was collected either via venipuncture or from a central venous catheter (CVC); a peripherally inserted central catheter (PICC), or an implanted central venous catheter (ICVC). Blood from age- and sex-matched healthy controls was collected via venipuncture. Venipunctures were performed aseptically by antecubital puncture via a 21-gauge needle. Blood was collected into vacuum tubes (1 volume 0.109 M trisodium citrate to 9 volumes blood) (VACUETTE, Greiner Bio-One). When collecting blood from a CVC, the catheter was first flushed with sodium chloride 0.9% and the first 20 mL of collected blood was discarded. Platelet-poor plasma (PPP) was prepared by double centrifugation at 2500 g for 15 min. Aliquots of 1 mL of the supernatant were frozen at −80°C until analysis.

### Reagents

Recombinant tissue factor (TF, Innovin) was from Siemens Healthineers. Recombinant tissue plasminogen activator (tPA, Actilyse) was obtained from Boehringer Ingelheim. Thrombomodulin (TM), phospholipids (20:20:60 mol% phosphatidylserine:phosphatidyl-ethanolamine:phosphatidylcholine), and α2-macroglobulin-plasmin complex (α2M-Pm; calibrator) were from Synapse Research Institute. Plasmin-specific fluorogenic substrate (Boc-Glu-Lys-Lys-AMC) was purchased from Bachem. The calibrator and reagents for the plasma TG assay were obtained from Stago (Diagnostica Stago). Anti-FDP single-domain antibody (sdAb) was obtained from Synapse Research Institute. 21 Polyclonal peroxidase-conjugated goat anti-human fibrinogen antibody was obtained from Rockland Immunochemicals Inc, and SuperSignal West Femto Maximum Sensitivity substrate was purchased from Thermo Fisher Scientific.

### Markers of Coagulation and Fibrinolysis

Plasma levels of plasminogen, fibrinogen, and α2-antiplasmin were measured in citrated plasma on the STA-R Max, according to the manufacturer’s recommendations (Diagnostica Stago). Plasma PAI-1 levels were measured with the Asserachrom PAI-1 kit, according to the manufacturer’s recommendations (Diagnostica Stago). Thrombin activatable fibrinolysis inhibitor (TAFI) activity levels were measured with the Pefakit TAFI, according to the manufacturer’s recommendations (Pentapharm).

FDPs were measured using an in-house developed enzyme-linked immunosorbent assay, as has been described. 21 In short, 384-well microliter plates (NUNC Maxisorp, Thermo Fisher Scientific) were coated overnight at 4°C with 2 μg/mL sdAb against FDP in a carbonate–bicarbonate coating buffer (pH: 9.6). Next, a blocking step was performed with 2% bovine serum albumin (BSA) and 0.05% Tween in phosphate-buffered saline (PBS) for 2 hours at room temperature (RT). After washing with 0.05% Tween in PBS (pH: 7.4), plasma samples (diluted 1:20 in PBS/1% BSA) were added and incubated for 2 hours at RT. Following another wash step, wells were incubated overnight at RT with polyclonal peroxidase-conjugated anti-fibrinogen goat antibody (1:10,000 dilution). Plates were then washed again before the addition of SuperSignal West Femto Maximum Sensitivity substrate. Luminescence was measured using the BioTek Synergy H1 multimode microplate reader (Biotek). The levels of FDP were expressed as a percentage of the level in normal pooled plasma (NPP), collected from healthy donors, and measured on the same plate.

### Clot Lysis Time

CLT in plasma was measured using a turbidimetric, kinetic method. 30 Samples were tested in duplicate and preheated for 10 min at 37°C. A total of 80 μL of plasma was incubated for 10 min at 37°C with 5 pM TF, 4 μM PL, and 0.2 μg/mL tPA in BSA-5 buffer (5 mg/mL BSA, 20 mM HEPES, 140 mM NaCl, and 0.02% NaN3, pH 7.35). Fibrin clot formation was started by adding 20 μL of a preheated CaCl _2_ /BSA-60 solution (final concentration 16.7 mM/60 mg/mL in 20 mM HEPES, 0.02% NaN3, pH 7.35). The optical density was measured at 405 nm every 30 s for 2 h at 37°C using a SpectraMax M2 plate reader (Molecular Devices). CLT was calculated as the time from half-maximal fibrin formation to half-maximal fibrin degradation with the Shiny app tool. 31

### Thrombin Generation Assay

TG was measured in PPP with the calibrated automated thrombinography (CAT) technique, as described previously. 32 33 To 20 μL of PPP reagent (TF in the presence of phospholipids), 80 μL of plasma were added and incubated for 10 min at 37°C. To this mixture, 20 μL of FluCa solution was added. The final concentrations in the well were 5 pM TF, 4 μM phospholipids, 16.7 mM CaCl _2_ , and 416.7 μM ZGGR-AMC. The Fluoroskan Microplate Fluorometer was used to measure fluorescent signals for 60 min. TG parameters were calculated by the Thrombinoscope software 5.0; lag time, time to peak (ttPeak), endogenous thrombin potential (ETP), and peak thrombin. The ETP and peak thrombin were expressed as a percentage of the levels in NPP, which was collected from healthy controls, and measured on the same plate. The inhibition of the ETP and thrombin peak by TM was calculated with the following formulas: [(ETP _TF_ − ETP _TF+TM_ )/ETP _TF_ ] × 100; [(Peak _TF_ − Peak _TF+TM_ )/Peak _TF_ ] × 100.

### Plasmin Generation Assay

PG was measured in PPP by cleavage of a plasmin-specific fluorogenic substrate, using a calibrated, automated method, modified from calibrated automated thrombography, as described previously (CT; Thrombinoscope). 27 28 34 Fibrinolysis was induced by combining 40 μL of 1:2 diluted plasma (100 μL plasma in 200 μL BSA-5 and 10 μL of a trigger solution [final concentrations: 5 pM TF, 4 μM phospholipids, 1.25 μg/mL tPA]) in a well. PG was measured in the presence and absence of TM with a final concentration of 2 nM (the concentration causing 50% inhibition of the peak height in pooled normal plasma) to test the sensitivity of the thrombin/TM TAFI pathway. Calibrator (α2M-Pm) and 1:2 diluted plasma were combined in the calibrator wells. Then, the well plate was incubated at 37°C in a Fluoroskan Microplate Fluorometer (Thermolabsystems OY), followed by automatic dispensing of 10 μL substrate solution (500 μM Boc-Glu-Lys-Lys-AMC, 16.7 mM CaCl2, BSA-60) to each well. After shaking the plate for 5 s, the fluorescence was measured every 20 s for 40 min. Samples were measured in duplicate. PG parameters were calculated by the Thrombinoscope software 5.0: lag time, ttPeak, endogenous plasmin potential (EPP), plasmin peak, and % inhibition of EPP and peak by TM. The EPP and peak were expressed as a percentage of the levels in NPP, collected from healthy controls and measured on the same plate. The inhibition of the EPP and peak by TM was calculated with the following formulas: [(EPP _TF_ − EPP _TF+TM_ )/EPP _TF_ ] × 100; [(Peak _TF_ − Peak _TF+TM_ )/Peak _TF_ ] × 100.

### Statistical Analysis

Statistical analyses were performed with SPSS version 28, and figures were generated with GraphPad Prism software version 8. Continuous data are presented as median and corresponding interquartile range (IQR, defined as the range between the 25th and 75th percentile of distribution). Categorical data are presented as numbers and percentages. The Mann–Whitney *U* test was used for comparison of continuous variables between unpaired groups. The Wilcoxon signed-rank test was used to compare continuous variables between paired groups. The chi-square or Fisher’s exact test, as appropriate, was used for comparison of categorical variables between groups. The Spearman test was used for correlation analysis. Associations between baseline hemostatic marker levels and the incidence of thromboembolic events were tested with univariable Cox proportional hazard models, treating thromboembolic events as a time-dependent covariate. Considering the small sample size of the study and the estimated number of thromboembolic events, potential associations were only analyzed with univariable models and not with multivariable models. Binary logistic regression analyses were performed to investigate whether PG differed between patients and controls when correcting for plasminogen levels. A two-sided *p* -value below 0.05 was considered statistically significant.

## Results

### Study Population

Between May 2022 and January 2023, 18 patients and 19 controls were enrolled. One of the 19 enrolled healthy controls was excluded due to being diagnosed with breast cancer shortly after enrollment. In Table 1 , baseline characteristics of the 18 included patients and 18 healthy controls are shown. The median age of the patients was 67 years, with 56% being female. At study inclusion, 61% of the patients were diagnosed with metastatic pancreatic cancer, and 39% with LAPC. In 72% of the patients, pancreatic cancer was newly diagnosed. The majority of the patients (66%) were treated with FOLFIRINOX, and 33% underwent treatment with nab-paclitaxel plus gemcitabine. None of the included patients received primary thromboprophylaxis during the follow-up period. Blood samples were collected via venipuncture in 28% of the patients, from a PICC in 11% of the patients, and from an ICVC in 61% of the patients. In the control group, blood was collected through venipuncture.

**Table 1:** Baseline characteristics of patients and controls.

Characteristic	Controls ( *n* = 18)	Patients ( *n* = 18)
Age, y (IQR)	63 (52–67)	67 (60–71)
Female, *n* (%)	9 (50)	10 (56)
BMI, kg/m ^2^ (IQR)	25 (23–28)	25 (21–27)
Comorbidities, *n* (%)		
▪ History of cardiovascular disease	2 (11)	2 (11)
▪ Coronary artery disease	0	2 (11)
▪ Congestive heart failure	0	1 (6)
▪ Peripheral artery disease	1 (6)	0 (0)
▪ Cerebrovascular disease	1 (6)	0 (0)
▪ Hypertension	1 (6)	5 (28)
▪ Hyperlipidemia	3 (17)	3 (17)
▪ Diabetes	0	2 (11)
▪ New-onset diabetes	0	1 (6)
Risk factors for VTE, *n* (%)		
▪ History of VTE	1 (6)	2 (11)
▪ BMI > 30 kg/m ^2^	2 (11)	2 (11)
Central venous catheter, *n* (%)	–	13 (72)
▪ PICC		2 (11)
▪ ICVC		11 (61)
WHO performance status, *n* (%)	–	
▪ 1		18 (100)
Diagnosis pancreatic cancer, *n* (%)	–	
▪ Newly diagnosed		13 (72)
▪ Recurrent disease		4 (22)
▪ Progressive disease		1 (6)
Stage pancreatic cancer, *n* (%)	–	
▪ Locally advanced		7 (39)
▪ Metastatic		11 (61)
Chemotherapy, *n* (%)	–	
▪ FOLFIRINOX		12 (66)
▪ Nab-paclitaxel/gemcitabine		6 (33)

Abbreviations: BMI, body mass index; FOLFIRINOX, folinic acid, fluorouracil, irinotecan, and oxaliplatin; ICVC, implanted central venous catheter; IQR, interquartile range; PICC, peripherally inserted central catheter; VTE, venous thromboembolism; WHO, World Health Organization.

Note: Median and interquartile ranges (25–75%) or number and percentage (%) are presented.

At the second time point ( *t*_8_ ), after 8 weeks of follow-up, blood samples were collected from 13 patients. Five patients were lost to follow-up due to death ( *n* = 3, 17%) or referral to their general practitioner for best supportive care ( *n* = 2, 11%).

### Markers of Coagulation and Fibrinolysis

When comparing baseline ( *t*_0_ ) coagulation and fibrinolytic markers between patients and controls, patients had higher levels of fibrinogen, FDP, and D-dimer ( Fig. 1 ). Elevated levels of FDP and D-dimer suggest an increase in in vivo coagulation and subsequent fibrinolysis. No significant difference was observed in plasma plasminogen, α2-antiplasmin, PAI-1, and TAFI levels when comparing patients at *t*_0_ and controls.

**Fig. 1:**
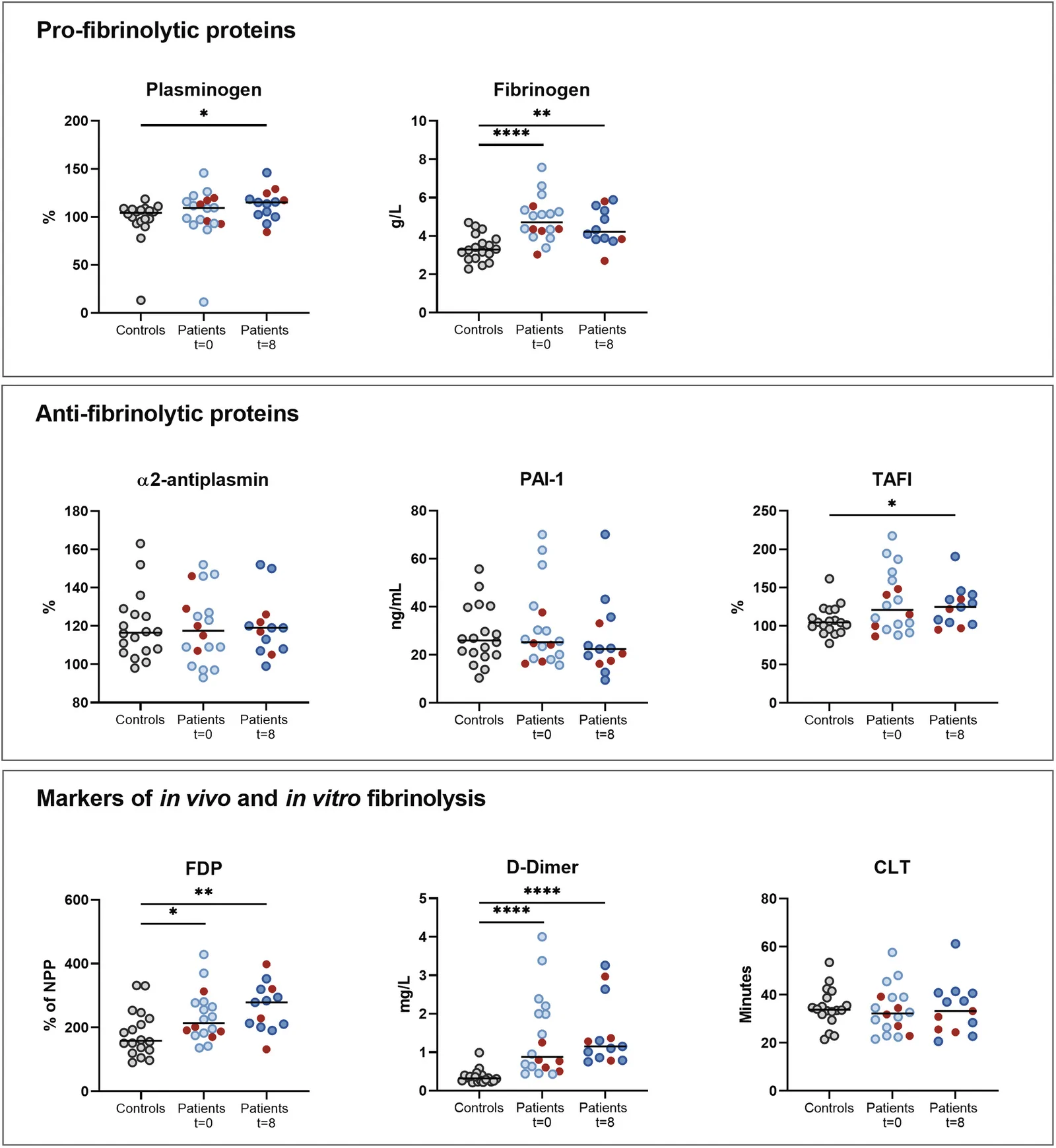
Plasma levels of coagulation and fibrinolysis markers in patients with pancreatic cancer and controls. Plasma levels of coagulation and fibrinolysis markers in pancreatic cancer patients before chemotherapy and 8 weeks after the start of chemotherapy and controls with a similar age and sex distribution. Plasma levels of fibrinogen (g/L), plasminogen (%), α2-antiplasmin (%), plasminogen activator inhibitor-1 (PAI-1; ng/mL), thrombin activatable fibrinolysis inhibitor (TAFI; %), fibrin degradation products (FDP; % from normal pooled plasma [NPP]), D-dimer (mg/L), and clot lysis time (CLT; minutes) are depicted. Medians are presented. Healthy controls are presented in gray, patients before chemotherapy ( *t* = 0) in light blue, and patients 8 weeks after the start of chemotherapy ( *t*_8_ ) in dark blue. Patients who developed a thromboembolic event, either venous or arterial, are presented in red. Wilcoxon signed–rank test or Mann–Whitney *U* tests were performed to compare the groups. * *p* < 0.05, ** *p* < 0.01, **** *p* < 0.0001.

When comparing patients before and during chemotherapy, plasma levels of plasminogen, fibrinogen, α2-antiplasmin, PAI-1, FDP, and D-dimer were not significantly different. However, a trend toward higher levels of plasminogen, TAFI, D-dimer, and FDP was observed. In addition, a trend toward a lower level of fibrinogen was observed at *t*_8_ . The plasminogen and TAFI levels in patients at *t*_8_ were significantly higher than the levels measured in the controls.

### Clot Lysis Time in Patients with Pancreatic Cancer and Healthy Controls

To explore the impact of pancreatic cancer on clot lysis, the CLT was assessed in pancreatic cancer patients and controls ( Fig. 1 ). No difference was observed when comparing patients at *t*_0_ to healthy controls (33.7 vs. 34.7 min, *p* = 0.563). Following 8 weeks of chemotherapy, the CLT (34.2 min) remained unchanged from the baseline measurement.

### Plasmin Generation in Patients with Pancreatic Cancer and Healthy Controls

The impact of pancreatic cancer on fibrinolysis was investigated with a novel method measuring PG. Patients with pancreatic cancer were found to have increased PG both before the initiation of chemotherapy ( *t*_0_ ) and 8 weeks thereafter ( *t*_8_ ), considering the higher EPP values ( Fig. 2 ; **Supplementary Table S1** , available in the online version only). The peak level of plasmin formation was higher in pancreatic cancer patients compared with controls at *t*_8_ . Additionally, a delay in PG was observed in pancreatic cancer patients at both *t*_0_ and *t*_8_ , characterized by a longer ttPeak PG. The lag time to PG was prolonged in patients at *t*_8_ ; however, no difference was found when comparing patients at *t*_0_ to healthy controls.

**Fig. 2:**
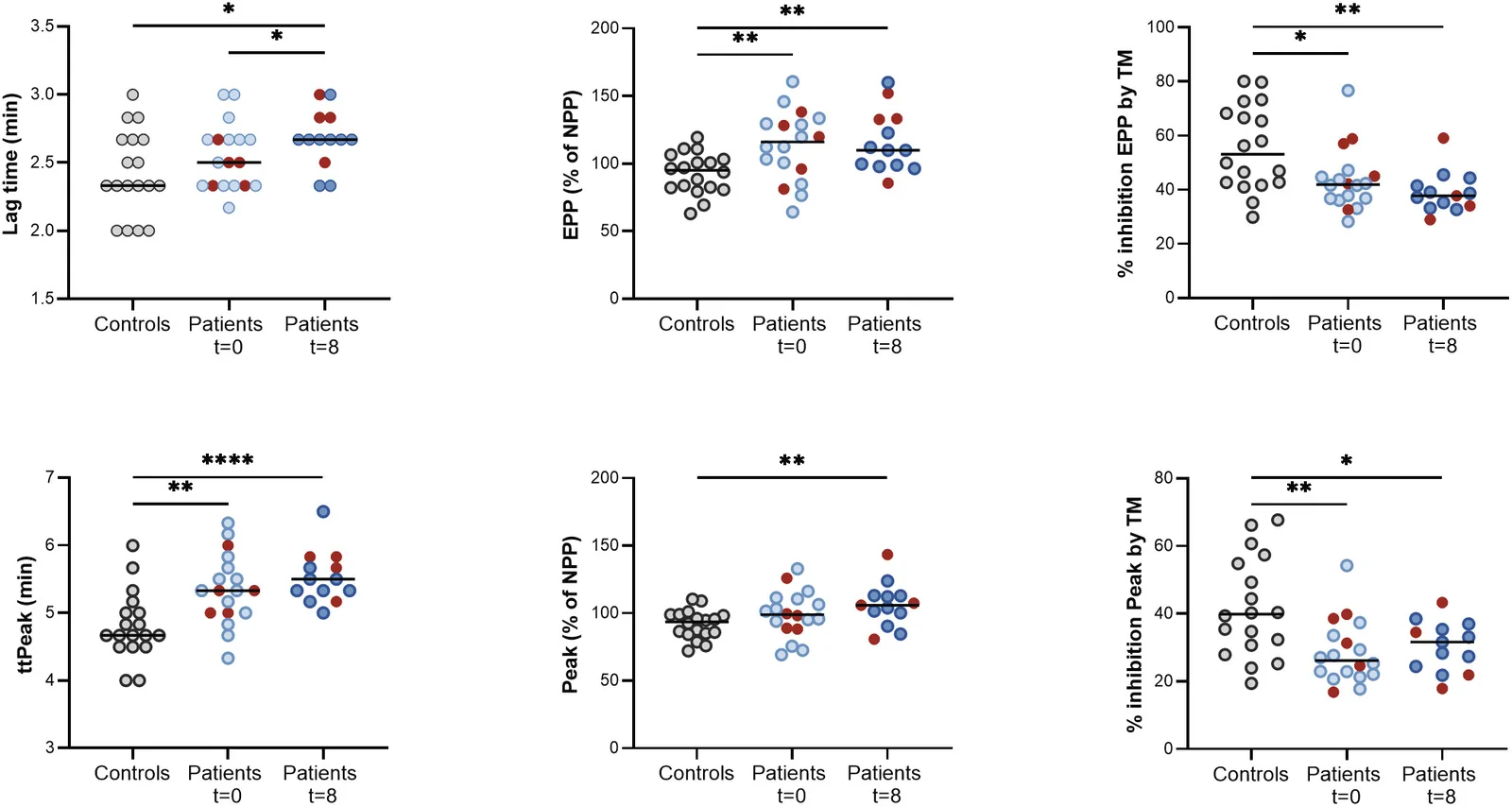
Plasmin generation in patients with pancreatic cancer and healthy controls. Plasmin generation parameters in patients with pancreatic cancer before chemotherapy and 8 weeks after the start of chemotherapy and matched controls. Plasmin generation was triggered with 1.25 μg/mL tPA and 5 pM TF in the presence or absence of TM in platelet-poor plasma. TM % inhibition represents the percentage reduction in EPP or plasmin peak after the addition of thrombomodulin relative to the corresponding value obtained in the absence of thrombomodulin, calculated as: [(value _− TM_ − value _+ TM_ )/value _−TM_ ] × 100. Higher values indicate greater inhibition by thrombomodulin. Medians are presented. Healthy controls are indicated in gray, patients before chemotherapy ( *t* = 0) are indicated in light blue, and patients during chemotherapy ( *t* = 8) are indicated in dark blue. Patients who developed a thromboembolic event, either venous or arterial, are indicated in red. Wilcoxon signed–rank test or Mann–Whitney *U* tests were performed to compare the groups. EPP, endogenous plasmin potential; TF, tissue factor; TM, thrombomodulin; *t*_0_ , first blood withdrawal before chemotherapy; *t*_8_ , second blood withdrawal 8 weeks after the start of chemotherapy. * *p* < 0.05, ** *p* < 0.01, *** *p* < 0.001.

Moreover, PG was evaluated in the presence of TM to test the thrombin/TM TAFI pathway. TM promotes the thrombin-mediated activation of TAFI. The percentage of inhibition of EPP and plasmin peak by TM was calculated. Pancreatic cancer patients were shown to have a reduced sensitivity to TM at both *t*_0_ and *t*_8_ , suggesting impaired TAFI-mediated inhibition of PG.

Comparison of PG in patients before ( *t*_0_ ) and during chemotherapy ( *t*_8_ ) revealed no significant changes in PG except for an increase in lag time at *t*_8_ .

### Relative Kinetics of Thrombin Generation and Plasmin Generation

PG is dependent on fibrin formation, which in turn relies on TG. To investigate whether the alterations in PG aligned with TG, TG was performed in plasma ( Table 2 ). Similar to PG, patients had a delay in TG before the start of chemotherapy ( *t*_0_ ) compared with controls, with a longer lag time and time to thrombin peak. No significant difference was found in the lag time and ttPeak thrombin when comparing patients during chemotherapy ( *t*_8_ ) to healthy controls. TG potential was similar between pancreatic cancer patients before chemotherapy and healthy controls. After 8 weeks of chemotherapy, the TG ETP and peak were lower in pancreatic cancer patients when compared with controls. The inhibition of the ETP by TM was higher in patients with pancreatic cancer at *t*_0_ than in healthy controls.

**Table 2:** Thrombin generation in patients with pancreatic cancer and healthy controls.

	Controls	Patients before chemotherapy, *t*_0_	Patients during chemotherapy, *t*_8_	*p* -Value
	*n* = 18	*n* = 18	*n* = 13	Control vs. *t*_0_
				Control vs. *t*_8_
				*t*_0_ vs. *t*_8_
Lag time (min)	2.9 (2.6–3.3)	3.7 (3.1–4.2)	3.3 (2.8–4.2)	**0.005**
				0.066
				0.422
ttPeak (min)	6.2 (5.7–6.8)	7.2 (6.0–7.8)	7.0 (6.0–7.7)	**0.040**
				0.091
				0.637
ETP (% of NPP)	110 (86–115)	100 (78–111)	82 (78–104)	0.203
				**0.028**
				**0.039**
Peak (% of NPP)	122 (116–144)	120 (97–137)	112 (97–120)	0.252
				**0.020**
				0.173
TM % inhibition ETP	41 (29–51)	50 (43–73)	45 (39–52)	**0.024**
				0.332
				0.075
TM % inhibition peak	24 (13–35)	31 (22–45)	25 (20–30)	0.074
				0.679
				0.101

Abbreviations: ETP, endogenous thrombin potential; NPP, normal pooled plasma; *t*_0_ , first blood withdrawal before chemotherapy; *t*_8_ , second blood withdrawal 8 weeks after the start of chemotherapy; TF, tissue factor; TG, thrombin generation; TM, thrombomodulin; ttPeak, time to peak.

Notes: Thrombin generation was triggered with 5 pM TF in platelet-poor plasma in the presence or absence of TM. Median and interquartile ranges (25–75%) are presented. Wilcoxon rank–sum tests or Mann–Whitney *U* tests were performed to compare the groups. Bold values indicate statistical significance ( *p* < 0.05). TM % inhibition represents the percentage reduction in ETP or thrombin peak after the addition of thrombomodulin relative to the corresponding value obtained with TF alone, calculated as: [(value _TF_ − value _TF + TM_ )/value _TF_ ] × 100.

To study whether PG aligned with TG, TG/PG ratios were calculated for every parameter ( Table 3 ). A decreased ratio between the TG ETP and PG EPP was found at *t*_0_ in patients with pancreatic cancer in comparison to controls. A lower ETP/EPP ratio indicates an increased PG capacity in proportion to TG capacity, revealing a net shift to a hyperfibrinolytic phenotype in patients with pancreatic cancer. Moreover, pancreatic cancer patients showed a higher TG to PG lag time ratio compared with healthy controls. This indicates that, in these patients, the delay in TG is disproportionately longer relative to the delay in PG.

**Table 3:** Thrombin generation to plasmin generation ratios in patients with pancreatic cancer and in healthy subjects.

	Controls	Patients before chemotherapy, *t*_0_	Patients during chemotherapy, *t*_8_	*p* -Value
	*n* = 18	*n* = 18	*n* = 13	Control vs. *t*_0_
				Control vs. *t*_8_
				*t*_0_ vs. *t*_8_
TG/PG lag time	1.16 (1.11–1.28)	1.37 (1.30–1.58)	1.25 (1.15–1.48)	**0.005**
				0.226
				**0.008**
TG/PG ttPeak	1.29 (1.17–1.45)	1.36 (1.10–1.52)	1.27 (1.12–1.33)	0.888
				0.489
				0.055
TG/PG ETP/EPP	1.17 (1.04–1.33)	0.84 (0.78–0.92)	0.78 (0.67–0.85)	**<0.001**
				**<0.001**
				0.101
TG/PG peak	1.44 (1.26–1.53)	1.21 (0.94–1.39)	1.02 (0.91–1.21)	**0.019**
				**<0.001**
				0.101
TG/PG inhibition ETP by TM	0.79 (0.65–0.94)	1.29 (0.82–1.67)	1.16 (0.98–1.54)	**0.002**
				**0.001**
				0.196
TG/PG inhibition peak by TM	0.61 (0.37–0.75)	1.29 (0.80–1.76)	0.81 (0.59–1.40)	**<0.001**
				0.094
				0.023

Abbreviations: EPP, endogenous plasmin potential; ETP, endogenous thrombin potential; PG, plasmin generation; *t*_0_ , first blood withdrawal before chemotherapy; *t*_8_ , second blood withdrawal 8 weeks after the start of chemotherapy; TM, thrombomodulin; TG, thrombin generation; ttPeak, time to peak.

Notes: To assess the alignment of thrombin generation and plasmin generation, TG/PG ratios were calculated for every parameter. Medians and interquartile ranges (25–75%) are presented. Wilcoxon rank–sum tests or Mann–Whitney *U* tests were performed to compare the groups. Bold values indicate statistical significance ( *p* < 0.05). For both thrombin generation and plasmin generation, TM % inhibition represents the percentage reduction in ETP, EPP, or peak after the addition of thrombomodulin relative to the corresponding value obtained in the absence of thrombomodulin, calculated as: [(value _−TM_ − value _+TM_ )/value _−TM_ ] × 100. The TG/PG inhibition ratios were calculated by dividing the TM-mediated inhibition of thrombin generation by the corresponding TM-mediated inhibition of plasmin generation. Higher inhibition values indicate greater sensitivity to thrombomodulin.

Additionally, a higher ratio of TG/PG ETP/EPP inhibition by TM was observed in patients with pancreatic cancer compared with controls, revealing a disbalance in the inhibition of coagulation and fibrinolysis by TM.

### Correlations of Plasmin Generation with Thrombin Generation, Coagulation, and Fibrinolysis Markers

Potential correlations between PG parameters and markers of in vivo coagulation and fibrinolysis were explored in patients before the start of chemotherapy and in healthy controls ( Fig. 3 ). In patients, a positive correlation was observed between plasminogen levels and the amount of generated plasmin, as indicated by the EPP and plasmin peak. This correlation was not evident in healthy controls. Moreover, in patients, plasminogen levels were also positively associated with the time to plasmin peak.

**Fig. 3:**
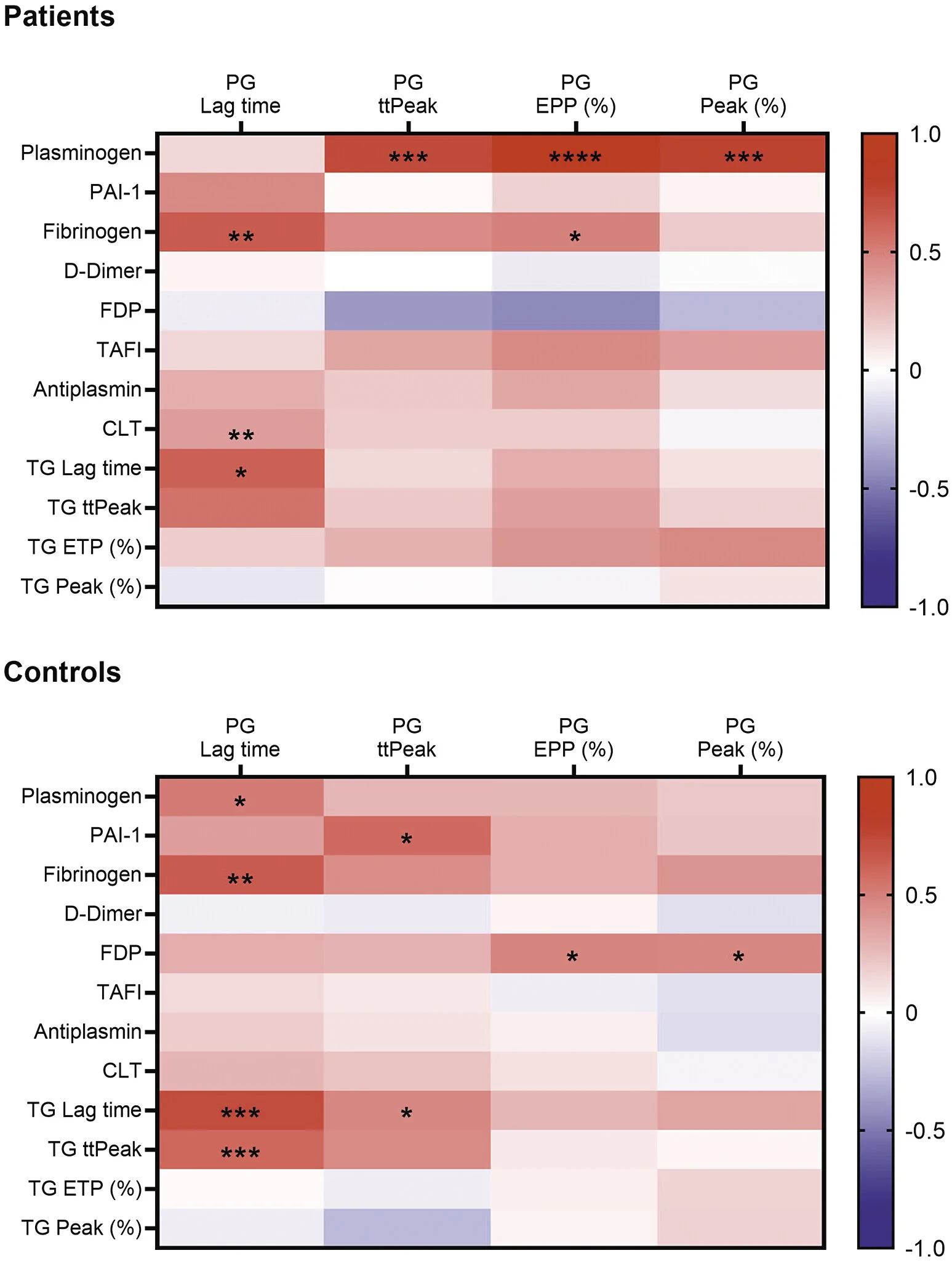
Heatmap of correlations between plasmin generation and coagulation and fibrinolysis markers in patients with pancreatic cancer and controls. Spearman correlation statistics are shown. In the upper rectangle, correlation statistics are shown for pancreatic cancer patients before the start of chemotherapy ( *t*_0_ ). In the lower rectangle, correlation statistics are shown for healthy controls. Positive correlations are presented in red; negative correlations are presented in blue. EPP, endogenous plasmin potential; ETP, endogenous thrombin potential; FDP, fibrin degradation products; LT, lag time; PAI-1, plasminogen activator inhibitor-1; PG, plasmin generation; TF, tissue factor; TG, thrombin generation; ttPeak, time to peak; VI, velocity index. * *p* < 0.05, ** *p* < 0.01, *** *p* < 0.001, **** *p* < 0.0001.

In both patients and controls, fibrinogen levels had a positive correlation with the lag time of PG. A positive correlation between fibrinogen levels and the PG EPP was found in patients, and not in controls.

When exploring correlations between PG and TG parameters, the lag time to PG was positively correlated with the lag time to TG in both patients and controls. This implies that the delay observed in PG is partially attributable to a delay in TG and subsequently fibrin formation.

### Correcting Plasmin Generation Capacity for Plasminogen Levels

As strong correlations between PG potential and plasminogen levels were observed, binary logistic regression analyses were performed to correct for plasminogen levels, with patient or control status as the dependent variable. When adjusting for plasminogen levels, EPP levels at baseline remained positively associated with pancreatic cancer (odds ratio [OR] = 1.055, 95% confidence interval [CI] = 1.013–1.098; **Supplementary Table S2** , available in the online version only). Moreover, plasmin peak was also positively associated with patient status (OR = 1.073, 95% CI = 1.013–1.137). These findings indicate that the increased PG capacity observed in patients with pancreatic cancer cannot be explained by higher plasminogen levels alone.

### Incidence of Thromboembolic Events and Plasmin Generation

PG at baseline ( *t*_0_ ) was compared between patients who did and did not develop a thromboembolic event, either venous or arterial ( Table 4 ; Fig. 2 ). No significant differences in PG parameters were demonstrated. A trend toward shorter lag time and increased TM-mediated inhibition of EPP and plasmin peak was observed in patients who developed a thromboembolic event.

**Table 4:** Plasmin generation and plasmin generation in relation to thrombin generation in patients who developed a thromboembolic event and patients who did not develop a thromboembolic event.

	Patients without thromboembolic event	Patients with a thromboembolic event	*p* -Value
	*n* = 13	*n* = 5	
Plasmin generation parameters, *t*_0_			
Lag time (min)	2.7 (2.3–2.8)	2.5 (2.3–2.6)	0.503
ttPeak (min)	5.3 (4.9–5.8)	5.3 (5.0–5.7)	0.849
EPP (% of NPP)	112 (93–132)	120 (89–133)	1.000
Peak (% of NPP)	102 (85–111)	98 (89–113)	0.849
Inhibition of EPP by TM (%)	42 (37–44)	45 (37–58)	0.289
Inhibition of peak by TM (%)	25 (22–32)	31 (21–39)	0.503
Thrombin generation to plasmin generation ratios, *t*_0_			
Lag time TG/PG	1.37 (1.30–1.57)	1.33 (1.26–1.65)	0.775
ttPeak TG/PG	1.34 (1.10–1.54)	1.37 (1.10–1.50)	1.000
ETP/EPP TG/PG	0.88 (0.80–0.98)	0.77 (0.64–0.81)	**0.046**
Peak TG/PG	1.22 (1.01–1.45)	0.94 (0.64–1.32)	0.289
Inhibition ETP/EPP by TM TG/PG	1.24 (0.97–1.72)	1.33 (0.73–1.62)	0.775
Inhibition peak by TM TG/PG	1.29 (0.78–1.74)	1.25 (0.65–1.82)	0.849

Abbreviations: EPP, endogenous plasmin potential; ETP, endogenous thrombin potential; min, minutes; PG, plasmin generation; *t*_0_ , before the start of chemotherapy; TG, thrombin generation; TM, thrombomodulin; ttPeak, time to peak.

Notes: Plasmin generation and TG/PG ratios, measured before the start of chemotherapy ( *t*_0_ ), in patients who developed thromboembolic events during 24 weeks of follow-up and patients who did not develop thromboembolic events. Bold values indicate statistical significance ( *p* < 0.05). Median and interquartile range (25–75%) are presented. The Mann–Whitney *U* test was used for group comparison. For both thrombin generation and plasmin generation, TM % inhibition represents the percentage reduction in ETP/EPP or peak after the addition of thrombomodulin relative to the corresponding value obtained in the absence of thrombomodulin, calculated as: [(value _−TM_ − value _+TM_ )/value _−TM_ ] × 100. Higher values indicate greater inhibition by thrombomodulin. TG/PG ratios were calculated by dividing each thrombin generation parameter by the corresponding plasmin generation parameter. For TG/PG inhibition ratios, the TM-mediated inhibition of thrombin generation was divided by the corresponding TM-mediated inhibition of plasmin generation.

When comparing TG/PG ratios, a significantly lower ETP/EPP ratio was found in patients who developed a thromboembolic event. This indicates that patients with incident thromboembolic events have an increased PG capacity in proportion to their TG capacity when compared with patients without thromboembolic events, consistent with a hyperfibrinolytic profile.

No significant time-dependent associations were found between PG parameters or TG/PG ratios and the development of thromboembolic events ( **Supplementary Table S3** , available in the online version only).

## Discussion

In this study, patients with metastatic pancreatic cancer and LAPC were shown to have an increased and delayed PG profile compared with healthy controls. To our knowledge, this is the first study measuring PG kinetics in patients with pancreatic cancer. Both before and during chemotherapy, patients with pancreatic cancer were found to have a higher EPP compared with healthy controls. A longer ttPeak PG was observed in patients with pancreatic cancer compared with controls. Furthermore, pancreatic cancer patients showed resistance to the inhibitory effects of TM on PG. Overall, the PG profile of pancreatic cancer patients remained largely unaffected by chemotherapy, except for a significantly longer lag time during chemotherapy.

FDP and D-dimer levels are the main markers of in vivo coagulation and subsequent fibrinolysis. In patients with pancreatic cancer, elevated levels of D-dimer and FDP have been reported in multiple studies and have been associated with mortality and response to palliative chemotherapy. 35 36 37 In line with previous studies, higher levels of the fibrinolysis end products FDP and D-dimer were observed in pancreatic cancer patients compared with healthy controls, indicating a state of hypercoagulation with subsequent hyperfibrinolysis. Moreover, higher levels of fibrinogen were observed in patients compared with controls. This observation is in line with other studies in patients with pancreatic cancer and other solid tumors, likely reflecting systemic inflammation, since fibrinogen functions as an acute-phase protein. 18 38 39 Plasminogen levels were only increased at the second time point, during chemotherapy, compared with controls. No difference was observed in the levels of the antifibrinolytic proteins α2-antiplasmin, PAI-1, and TAFI when comparing pancreatic cancer patients before chemotherapy to healthy controls. After 8 weeks of chemotherapy, TAFI levels were increased in pancreatic cancer patients when compared with controls.

PAI-1 is a key inhibitor of the fibrinolytic system, blocking the activity of plasminogen activators tPA and urokinase-type plasminogen activator (uPA). Previous studies have reported elevated PAI-1 levels in patients with pancreatic cancer and found an association of higher PAI-1 levels with VTE incidence. 22 23 In contrast, no difference in plasma PAI-1 levels was observed between patients and controls in the present study. 23 A possible explanation is the known circadian variation in PAI-1 levels, which may have influenced our results, as blood samples were collected during routine clinical visits without time-of-day standardization. 23 40 Furthermore, PAI-1 functions as an acute-phase protein, with levels increasing in response to inflammation. 41 Differences in the inflammatory status of the patient populations across studies may also explain the variation in PAI-1 levels.

PG depends on fibrin formation, as fibrin is not only a substrate for plasmin but also a cofactor for tPA-mediated plasminogen activation. 42 To evaluate the balance between fibrinolysis and coagulation, we examined PG in relation to TG. In patients with pancreatic cancer, a disbalance between PG and TG was observed, with a higher amount of plasmin being generated in relation to thrombin, when compared with healthy subjects. Moreover, TM inhibited TG more effectively than PG in these patients. As a result, TM further amplified this shift toward a phenotype characterized by increased PG relative to TG, suggestive of enhanced fibrinolytic potential.

Potential correlations were explored between PG parameters and markers of in vivo coagulation and fibrinolysis. In patients, positive correlations were identified between plasminogen levels and the EPP and peak plasmin, whereas these correlations were not present in controls. Because plasminogen levels showed greater variability and were generally higher in patients with pancreatic cancer, these differences could partially influence the strength of these correlations. Importantly, after correcting for plasminogen levels, PG potential remained associated with patient status, indicating that the increased PG potential is not solely due to altered plasminogen levels. In both patients and controls, higher fibrinogen levels correlated with a longer lag time to PG and time to plasmin peak. This finding aligns with our previous work showing that fibrinogen levels are also positively correlated with TG lag time in patients with pancreatic cancer. 21 The delay in PG can be partly explained by the delay in TG. The prolongation in lag time with higher fibrinogen levels was previously reported 43 and is supported by comparison of TG in normal versus defibrinated plasma. 44 This delay is thought to result from the interaction of fibrin(ogen) with thrombin, which inhibits thrombin-mediated factor VIII activation. 43 45 46

Another commonly used and more traditional method for assessing fibrinolysis is the CLT assay. In the present study, CLT did not differ between patients with pancreatic cancer and healthy controls, suggesting a normal fibrinolytic function. This finding appears to contrast with the increased PG capacity observed in this same patient group. However, it is important to note that the CLT and PG assays measure different aspects of the fibrinolytic process. While PG assesses the capacity to generate plasmin—capturing the early phase of fibrinolysis and its reliance on plasminogen and fibrinogen—the CLT measures the overall time needed for both clot formation and subsequent breakdown. Our results are consistent with those of Risman et al, who similarly reported no difference in CLT when comparing 17 patients with pancreatic cancer to seven healthy individuals. 47

PG parameters were compared between patients who developed a thromboembolic event, either venous or arterial, and patients who did not. No significant differences in individual PG parameters were observed. When examining the TG to PG ratios, patients who developed a thromboembolic event had a lower TG/PG ETP/EPP ratio at baseline. However, given the limited number of thromboembolic events, this finding should be considered exploratory. Furthermore, the TG/PG ETP/EPP ratio was not significantly associated with thromboembolic events during follow-up (hazard ratio = 0.022, *p* = 0.125).

The principal function of the plasminogen activation system is the generation of plasmin to degrade the fibrin framework of blood clots. However, its components contribute to multiple biological processes beyond fibrinolysis. Clinical studies have shown that fibrinolytic proteins are associated with cancer progression, metastasis, and survival, indicating a role for the fibrinolytic system in tumor biology. 48 Plasmin promotes metastasis by degrading basement membrane components, releasing tumor growth factors from the extracellular matrix, and activating matrix metalloproteinases. 48 49 50 51 52 53 54 55 In patients with cancer who develop VTE, higher circulating levels of the plasminogen activator tPA have been reported. 56 Tumor growth, tumor invasion, and angiogenesis have been shown to be affected by tPA in pancreatic cancer cell lines. 57 In patients with pancreatic cancer, uPA has been shown to be overexpressed in tumor tissue, and increased uPA expression has been associated with metastasis, increased cancer stage, and worse survival. 58 59 60 61 62 The binding of uPA to its receptor (uPAR) enhances cell-surface fibrinolysis, and patients with pancreatic cancer display increased circulating levels of soluble uPAR compared with those with benign pancreatic lesions. 63 Experimental mouse models further support a tumor-promoting role for profibrinolytic proteins: plasminogen-deficient mice show reduced pancreatic tumor growth and metastatic capacity. 64 Additionally, an anti-uPAR antibody reduced tumor size, invasion, and metastasis in a pancreatic cancer mouse model. 65 The increased PG potential observed in the present study may therefore reflect cancer-related upregulation of tPA and uPA levels. Extracellular vesicles may also contribute to the altered PG phenotype observed in pancreatic cancer. While these vesicles are best known for their procoagulant properties, recent evidence suggests that they can also modulate fibrinolytic pathways and PG. 66 In particular, extracellular vesicles have been shown to provide a surface that supports uPA/uPAR-mediated plasminogen activation and subsequent PG. 66 In addition, extracellular vesicles derived from endothelial cells may carry fibrinolytic proteins such as tPA and uPA. 66 As pancreatic cancer is associated with increased release of tumor-derived extracellular vesicles, these particles may represent an additional mechanism underlying the enhanced PG observed in our cohort. 67 Further studies are needed to determine whether PG potential is associated with disease progression and survival in patients with pancreatic cancer.

Our study has limitations that should be acknowledged. The primary limitation is the small sample size of 18 patients and 18 controls. This number was predetermined to enable characterization of novel coagulation assays in patients with pancreatic cancer compared with healthy individuals. 21 Despite the sample size, significant differences in PG kinetics were observed between patients and controls. However, no significant time-dependent associations between PG parameters and thromboembolic events were identified. A larger cohort study is required to investigate these potential associations. A second limitation is the varying methods of blood withdrawal within the patient group and between patients and controls. To minimize patient burden, an extra blood tube was collected during routine clinical blood draws. It has been theorized that the method of blood withdrawal influences contact activation and hence TG. 68 To our knowledge, there is no literature available on the potential effects of blood withdrawal methods on PG capacity. In future cohort studies, it would be optimal to standardize the method of blood withdrawal. Third, PG was compared at two time points for only 13 of the 18 patients. For the remaining five patients, follow-up sampling was not possible due to mortality or transition to best supportive care. This may have introduced attrition bias, as the patients lost to follow-up could have had different PG profiles. Lastly, as PG was measured in plasma, blood cell–dependent effects were not measured. 21

In conclusion, patients with locally advanced and metastatic pancreatic cancer exhibited an altered PG phenotype compared with controls, characterized by delayed and increased PG. These findings were observed both before and during chemotherapy, suggesting that this phenotype remains largely preserved during treatment. The prolonged lag time may reflect alterations in the initiation of PG, whereas the increased peak and EPP indicate an enhanced capacity to generate plasmin. Given the limited sample size and the small number of thromboembolic events, the clinical implications of these findings remain to be established. Further studies in larger cohorts are warranted to validate these observations and to determine whether PG parameters are associated with thromboembolic risk and clinical outcomes. The ongoing “Predicting Venous Thromboembolism in Patients with Pancreatic Cancer” (SENEPANC) study, a prospective, multicenter cohort study coordinated by the Dutch Pancreatic Cancer Group, will further explore the potential value of PG parameters as biomarkers of thromboembolic risk in patients with locally advanced and metastatic pancreatic cancer.

***What is known about this topic?***

- Patients with pancreatic ductal adenocarcinoma are at high risk of thromboembolic complications.
- While hypercoagulability has been extensively studied, global assessment of fibrinolytic capacity in pancreatic cancer remains limited.
- Conventional fibrinolysis assays provide limited insight into the dynamics of plasmin generation.

***What does this paper add?***

- This is the first study to characterize plasmin generation kinetics in patients with pancreatic ductal adenocarcinoma using a calibrated automated plasmin generation assay.
- Patients with pancreatic cancer exhibit a delayed but increased plasmin generation capacity, indicating a disbalance between coagulation and fibrinolysis.
- Plasmin generation remained largely unchanged during chemotherapy.

## Supplementary Material

Ergänzendes Material Supplementary Material
