# Supplementary material for: Plasmin Generation Kinetics Reveal a Hyperfibrinolytic Phenotype in Patients with Pancreatic Ductal Adenocarcinoma
Source: TH Open. 2026 Sep 15;10:a29256824. doi: 10.1055/a-2925-6824 (PMC13643898; doi:10.1055/a-2925-6824)
Supplement: Supplementary file 1 [file 10-1055-a-2925-6824_29422307.pdf]

Supplemental Material

SUPPLEMENTARY TABLE 1 | Plasmin generation in patients with pancreatic cancer and healthy controls

	**Controls** n=18	**Patients before chemotherapy, t_0** n=18	**Patients during chemotherapy, t_8** n=13	**P-value** Control vs. t_0<br>Control vs. t_8<br>t_0 vs. t_8
Lag time (min)	2.3 (2.3-2.7)	2.5 (2.3-2.7)	2.7 (2.6-2.8)	0.265<br>**0.021**<br>**0.035**
ttPeak (min)	4.7 (4.5-5.0)	5.3 (5.0-5.7)	5.5 (5.3-5.7)	**0.003**<br>**<0.001**<br>0.156
EPP (% of NPP)	95 (82 -104)	116 (93-131)	110 (98-133)	**0.008**<br>**0.004**<br>0.701
Peak (% of NPP)	94 (85-99)	99 (89-111)	106 (95-113)	0.249<br>**0.006**<br>0.311
Inhibition EPP by TM (%)	55 (42-69)	42 (37-46)	38 (34-43)	**0.017**<br>**0.001**<br>0.221
Inhibition Peak by TM (%)	40 (30-55)	26 (22-35)	32 (23-36)	**0.003**<br>**0.028**<br>0.345

Plasmin generation was triggered with 1.25 µg/mL tPA and 5 pM TF, with or without 2 nM TM in PPP. Median and interquartile ranges (25%-75%) are presented. The Mann-Whitney U test was used for comparison of continuous variables between unpaired groups. The Wilcoxon signed rank test was used to compare continuous variables between paired groups (t_0 and t_8). EPP: Endogenous plasmin potential; PPP: platelet poor plasma; TF: tissue factor; TM: thrombomodulin; t_0: first blood withdrawal before chemotherapy; t_8: second blood withdrawal 8 weeks after the start of chemotherapy.

SUPPLEMENTARY TABLE 2 | Associations of plasmin generation capacity and plasminogen levels with pancreatic cancer patient status

Variable	Univariate	
	**OR (95% CI)**	**p-value**
EPP, % of NPP	1.049 (1.009-1.089)	0.015
Peak, % of NPP	1.040 (0.988-1.095)	0.132
Plasminogen, %	1.011 (0.982-1.040)	0.466
	**Multivariate model 1**	
	**OR (95% CI)**	**p-value**
EPP, % of NPP	1.055 (1.013-1.098)	0.009
Plasminogen, %	1.000 (0.972-1.030)	0.974
	**Multivariate model 2**	
	**OR (95% CI)**	**p-value**
Peak, % of NPP	1.073 (1.013-1.137)	0.016
Plasminogen, %	1.002 (0.974-1.031)	0.897

Binary logistic regression analyses were performed to correct PG capacity for plasminogen levels, with patient or control status as the dependent variable. CI: confidence interval; EPP: endogenous plasmin potential; NPP: normal pooled plasma; OR: odds ratio.

SUPPLEMENTARY TABLE 3 | Time-dependent associations between plasmin generation and the development of thromboembolic events

Variable	Univariate	
	**HR (95% CI)**	**p-value**
**Plasmin generation**		
Lag time, min	0.350 (0.008-15.258)	0.586
Time to peak, min	0.946 (0.148-6.051)	0.953
EPP, % of NPP	0.996 (0.962-1.032)	0.828
Peak, % of NPP	0.996 (0.941-1.055)	0.892
Inhibition of EPP by TM, %	1.021 (0.961-1.084)	0.500
Inhibition of Peak by TM, %	1.011 (0.928-1.101)	0.804
**Thrombin generation to plasmin generation ratios**		
TG/PG Lag time	0.778 (0.023-26.572)	0.889
TG/PG Time to peak	0.785 (0.021-27.696)	0.884
TG/PG ETP/EPP	0.022 (0.000-2.869)	0.125
TG/PG Peak	0.288 (0.033-2.533)	0.262
TG/PG Inhibition by TM ETP/EPP	0.708 (0.136-3.690)	0.682
TG/PG Inhibition of Peak by TM	0.927 (0.297-2.889)	0.895

Univariate Cox regression analyses were performed to study the time-dependent association between PG parameters, TG/PG parameter ratios, and thromboembolic events. CI: confidence interval; EPP: endogenous plasmin potential; ETP: endogenous thrombin potential; HR: hazard ratio; TM: thrombomodulin.
